# Evaluating stakeholder involvement in building a decision support tool for NHS health checks: co-producing the WorkHORSE study

**DOI:** 10.1186/s12911-020-01205-y

**Published:** 2020-08-10

**Authors:** Ffion Lloyd-Williams, Lirije Hyseni, Maria Guzman-Castillo, Chris Kypridemos, Brendan Collins, Simon Capewell, Ellen Schwaller, Martin O’Flaherty

**Affiliations:** 1 Department of Public Health and Policy. Institute of Population Health Science, University of Liverpool, The Quadrangle, University of Liverpool, Liverpool, L69 3GB UK; 2 Department of Social Research, University of Helsinki, Helsinki, Finland

**Keywords:** Co-production, Stakeholder engagement, Group model building, NHS health checks

## Abstract

**Background:**

Stakeholder engagement is being increasingly recognised as an important way to achieving impact in public health. The WorkHORSE (Working Health Outcomes Research Simulation Environment) project was designed to continuously engage with stakeholders to inform the development of an open access modelling tool to enable commissioners to quantify the potential cost-effectiveness and equity of the NHS Health Check Programme.

An objective of the project was to evaluate the involvement of stakeholders in co-producing the WorkHORSE computer modelling tool and examine how they perceived their involvement in the model building process and ultimately contributed to the strengthening and relevance of the modelling tool.

**Methods:**

We identified stakeholders using our extensive networks and snowballing techniques. Iterative development of the decision support modelling tool was informed through engaging with stakeholders during four workshops. We used detailed scripts facilitating open discussion and opportunities for stakeholders to provide additional feedback subsequently. At the end of each workshop, stakeholders and the research team completed questionnaires to explore their views and experiences throughout the process.

**Results:**

30 stakeholders participated, of which 15 attended two or more workshops. They spanned local (NHS commissioners, GPs, local authorities and academics), third sector and national organisations including Public Health England.

Stakeholders felt valued, and commended the involvement of practitioners in the iterative process. Major reasons for attending included: being able to influence development, and having insight and understanding of what the tool could include, and how it would work in practice. Researchers saw the process as an opportunity for developing a common language and trust in the end product, and ensuring the support tool was transparent. The workshops acted as a reality check ensuring model scenarios and outputs were relevant and fit for purpose.

**Conclusions:**

Computational modellers rarely consult with end users when developing tools to inform decision-making. The added value of co-production (continuing collaboration and iteration with stakeholders) enabled modellers to produce a “real-world” operational tool. Likewise, stakeholders had increased confidence in the decision support tool’s development and applicability in practice.

## Background

Assessing which interventions are most effective, cost-effective and equitable are difficult in public health due to its existence in a complex dynamic system. Dynamic simulation models are increasingly used within the health sector, including public health to optimise decisions in limited resource settings [1]. Therefore, they can be used to model potential interventions and their outcomes to help inform decision-making. However, uptake of ‘return on investment’ type tools by decision-makers is low. Whilst stakeholder engagement in conceptual model building is well established [2, 3], and studies to explore why stakeholders do not use simulation models [4–6] exist, studies describing active engagement with stakeholders during computational model building are lacking. Research Councils are increasingly encouraging researchers to consider the wider impact of their research. As part of a strategy to ensure research is of benefit and relevance in the “real world” beyond academia and has the greatest impact to the end-user, stakeholder engagement is a key component in public health research [7, 8].

The concept of stakeholder theory was originally described by Freeman (1984) [9]. Freeman described the role of stakeholder theory in relation to organisational business management and stressed the interrelated relationships between a business and its customers, suppliers, employees, investors, communities and others who have a stake in the organization. Stakeholder engagement has been adapted, applied and increasingly used in the field of health research. Boaz (2018) [10] identifies three key principles for stakeholder engagement in health research: 1. Organisational (e.g. clear objectives, resources, inclusion of key stakeholders); 2. Values (e.g. commitment of project team, shared understanding and commitment); 3. Practices (e.g. flexibilty within the research process, systematic data gathering, involvement of stakeholders as an iterative process). In the context of the WorkHORSE (Working Health Outcomes Research Simulation Environment) project, we defined and identified stakeholders as individuals and organisations who would be the commissioners and users of the WorkHORSE modelling tool to make decisions regarding increasing the uptake of the NHS Health Check Programme based upon effectiveness, cost-effectiveness and equity). However, we also acknowledge that there will be a wider group of beneficiaries of the modelling tool including the computer modelling community, health professionals involved in the delivery and uptake of NHS Health Checks, and specific members of the public who would benefit from a more targeted approach, ensuring the best possible health outcomes.

Various stakeholder engagement approaches exist, including questionnaires, interviews, focus groups, conferences, forums and symposia, nominal group technique and Delphi techniques [9, 11] The objective of these approaches also varies from canvassing and reflecting a range of opinions, to building consensus or providing expert opinion to fill gaps in the evidence. A review by Mallery et al. [12] identified various innovative methods including online, collaborative forums and online communities. Stakeholder engagement ideally aims to establish and maintain involvement throughout the research process and maintain interactivity between researchers and users [13].

The level of stakeholder involvement employed can vary depending upon the objectives of the engagement and outcomes to be achieved [14]. The Organisation for Economic Co-operation and Development [15] distinguishes six levels of stakeholder engagement depending on the processes and the intentions they pursue. The processes range from the least engaged to most engaged: communication (sharing information to increase knowledge), consulation (gathering information and experience of stakeholders without obligation to use in fiinal outputs), participation (taking part in the project process, but not the decision making process), representation (structural level of engagement), partnerships (agreed upon collaboration), co-production (balanced share of power). Co-production was chosen for the WorkHORSE project as it was imperative to deliver a modelling tool that would be accessible, comprehensive, and would ultimately lead to more effective NHS Health Check uptake and delivery.

In its broadest sense, co-production is defined as a collaboration in governance, priority-setting, conducting research, knowledge translation, which involves researchers and others with a stake in the project [16]. In public health research these can include public, patients, health care providers, and decision makers and policy-makers. The key component is joint decision-making [17]. In the context of this study, joint decision-making referred to the research team and stakeholder participants, but did not include patients. Co-production is regarded as a positive component of research development and delivery. However, experts in the field have highlighted the potential challenges and complexity of co-production. Doing co-production properly can be expensive financially and in terms of time. Coproduction as an aim of research needs to consider the added value and usefulness in helping the research meet its desired outcome. For example, ensuring that the correct mix of stakeholders are involved, at the appropriate stages of the research process, ensuring joint decision making is managed and ensuring that coproduction supports, rather than mitigates against the quality of the research [18].

Based upon the nature of the WorkHORSE project, to develop a decision support tool for commissioners of the NHS Health Check Programme (NHSHCP), we developed a series of workshops utilising the co-production approach. WorkHORSE was a two-year project designed to continuously engage with stakeholders, via four iterative workshops, to coproduce an open-source/open-access web-based modelling tool to enable commissioners to quantify the potential cost-effectiveness and equity of the NHS Health Check Programme in England. The NHSHCP is offered at five-year intervals to all adults aged 40–74 years who do not have pre-existing vascular conditions. The programme aims to prevent cardiovascular disease together with detecting diabetes and chronic kidney disease and raising awareness of dementia. The programme includes cardiovascular disease (CVD) risk stratification and people identified as being high-risk are offered appropriate treatment including behavioural change interventions, statins for high cholesterol and antihypertensive drugs for high blood pressure [19].

In our study, coproduction involved the research project team (modellers, researchers and lay representatives) and stakeholders (i.e. individuals involved in the assessment, commissioning and delivery of NHS Health Checks [19] (https://www.healthcheck.nhs.uk), a cardiovascular disease prevention programme delivered by Local authorities). Through a series of four interrelated workshops, a web-based decision support tool was developed via an interactive and iterative process whereby the research team and stakeholders collaborated through all phases of the research project.

**The WorkHORSE project had five key aims: 1**. Co-produce proposals with stakeholders to inform the desirable features of the user-friendly model and identify additional locally relevant scenarios to test. **2.** Update the evidence base to support model and scenario development. **3**. Further develop our computational model to allow for developments and changes to NHS HCP and the diseases it addresses. **4**. Assess the effectiveness, cost-effectiveness and equity of alternative strategies for NHS HCP implementation. and **5**. Propose a sustainability and implementation plan to deploy our user-friendly computational model at the local level.

The key objective was to evaluate the involvement of stakeholders in co-producing the WorkHORSE computer modelling tool and examine how they perceived their involvement in the model building process and ultimately contributed to the strengthening and relevance of the modelling tool.

In this paper, we describe the experience of the modellers, stakeholders and lay advisers in shaping and informing the development of the model and the added value of co-production.

## Methods

### Research participants

#### Stakeholder mapping and recruitment

We developed a stakeholder recruitment grid based on our extensive public health networks at the local, regional and national level. The WorkHORSE project team identified relevant organisations, who were actively involved in the commissioning/delivery of NHS Health Checks and/or had a vested interest in development of the modelling tool and the impact it would have on the NHSHCP Individuals from these organisations were added to the recruitment grid. The final recruitment grid contained a diverse group of stakeholders from different organisations including Public Health England (national and regional level), British Heart Foundation, Diabetes UK, Alzheimer’s UK, NICE (The National Institute for Health and Care Excellence), BMA (British Medical Association), Alcohol Research UK, North West Strategic Clinical Network, Director of Public Health, Local Government Association, Clinical Commissioning Groups (CCGs), Local Authorities (LAs), GPs, Pharmacies and Academics. Inviting a cross-section of stakeholders representing local, regional and national perspectives provided a broad skillset and perspective in the process of co-producing the tool. Stakeholders were sent an email invitation to attend the workshops. If specific stakeholders were unable to attend, we used snowballing techniques to identify other associated individuals at their organisation to invite. Depending on the objectives of the workshop, we would either deliberately have tables with a broad mixture of perspectives (HCP, local, regional, national decision makers) or would sit people from similar organisations on the same table.

#### Lay adviser recruitment and involvement

Four lay advisers were recruited via the National Institute for Health Research Patient and Public Involvement Network and local Healthwatch. In the context of the WorkHORSE project, the lay advisers were part of the research team (i.e. Patient and Public Involvement in research, (PPI). Furthermore, they also provided us with useful insights into their experience of being involved in the co-production process. All the lay advisers had a personal experience of using Health Checks and were interested in how the development of the decision support tool would benefit health check provision. The lay advisers assisted the research team with the project application, provided advice and support with the delivery of the project, and ensured the perspective of the public was represented by helping us to write clear, understandable literature for engagement with the public, and dissemination within the research community from the lay perspective.

#### The modelling team

It was imperative to evaluate the modelling team experience of the co-production process in order to provide context to the stakeholders’ experience. The four computer modellers were all team members of the NCD Prevention and Food Policy Research Group, Department of Public Health and Policy, University of Liverpool. All had previous experience of developing computer decision support tools for end users.

### Workshop design

The design of the workshop programme was theory-based using the Cairney/Oliver key co-production principles [18, 20]. This included co-identifying the requirements of the decision support tool based upon stakeholders’ current views and experience and future requirements; to work iteratively over the lifespan of the project to co-steer the decision support tool content and outputs, and to co-develop interpretations of the decision support tool and implications for dissemination and end-use.

These events were carefully planned using a “script approach”, an example is provided in Appendix 1, with the scripted approach for Activity 1, Workshop 1 shown in Table 1. We adapted previously validated scripts to our specific needs and context in order to structure and gently facilitate the worshop process (Scriptapedia based upon the work of Hovmand et al. (2012) [21] group model building (GMB) approach as part of a general framework. GMB is a participatory method for involving people in designing, creating or validating system dynamics models. GMB consists of one or more sessions (workshops) with a carefully selected group of stakeholders and the use of small structured exercises with specific objectives and outputs; and the extensive use of facilitation, discussions and analysis. These activities enabled the team to engage with stakeholders in the co-design of the decision support tool, facilitating open discussion and opportunities for stakeholders to provide additional feedback subsequently.

**Table 1:** Excerpt of scripted approach detailed in appendix 1

**ACTIVITY 1** *To identify stakeholder’s expectations of the WorkHORSE health checks project; To identify what is working well and not so well and future hopes for the NHS Health Checks Programme (NHS HCP)* **Purpose:** To elicit what is working well and not so well and future hopes for the NHS HCP **Time:** 25 min for the activity; 25 min feedback from individuals and group discussion **Materials:** 1. Flipchart paper with headings (*what is working well, not so well and future hopes)* for each table of participants 2. Three different colours of post it notes for each participant 3. Back felt tip pens for each stakeholder 4. Masking tape 5. Tape recorder to record individual/group feedback/discussion **Outputs:** List of stakeholder’s comments regarding what is working well and not so well and future hopes for NHS HCP. **Roles:** 1. Facilitator with knowledge of the topic 2. Scribe/recorder to document the session **Steps:** 1. Participants are given several sheets of paper in each colour. The facilitator explains that they will be writing what they believe works well and not so well and future hopes relating to NHS HCP and then sharing them with the group. 2. The facilitator states which colour post it note represents working well; not working well; future hopes, and to stick responses onto the appropriate piece of flipchart paper (with headings) 3. Stakeholders have 25 min for the whole task (5 min to write down individual thoughts, 20 min to discuss with other group members) 4. Each stakeholder reads out one aspect working well, one not so well and future hope for NHS HCP. After each participant has shared once, facilitator opens the floor to all participants for any further comments about what is working well and not so well, challenges and future hopes and opportunity for discussion. (25 min) 5. Facilitator then tries to identify some of the themes and summarises. (10 min) 6. Scribe/recorder records all comments regarding what is working well and not so well and future hopes in the session notes. 7. ALL flipchart paper to be collected from each table and either stuck up at the back of the room or laid on tables at the back of the room – stakeholders are encouraged to add additional comments at any time to the flipchart papers.	

The WorkHORSE project workshops had the overall aim of co-producing the web-based decision support tool with stakeholders. Each included a series of small-group exercises with specific objectives and outputs. The workshops were iterative in their approach and involved an independent facilitator in their delivery. Each workshop used specific questions and activities. Immediate feedback was obtained via Post-it Notes, flipcharts, small group and plenary discussions (Table 2).

**Table 2:** Summary of Workshop Aims and Activities

Workshop	Aims	Activity
1	• build a mutual working relationship between stakeholders and the research team; • ensure an understanding of the health checks process for scenario building • generate rich and valuable interaction to inform model development	*Activity 1:* Groupwork to identify stakeholder’s expectations of the WorkHORSE health checks project and identify what is working well and not so well and future hopes for the NHS HCP (heterogeneous grouping) *Activity 2:* Groupwork to elicit what features/specifications will make WorkHORSE a useful tool for the stakeholders (homogenous grouping)
2	• develop the priorities for the model functionality • explore how alternative implementations of health checks could be modelled through the input parameters of the tool • clear specification on outputs/visualisations in terms of immediate accessibility required by stakeholders	*Activity 1:* Groupwork to enable stakeholders to consider alternative HC implementations and practice modelling these implementations leading to a blueprint for co-produced scenario(s) (heterogeneous grouping) *Activity 2:* Groupwork to rank the importance of model outputs and visualisations which will make WorkHORSE a useful tool for stakeholders (homogenous grouping)
3	• focus on the design and co-production of realistic model scenarios (previously raised by stakeholders) • explore model outputs and confirm their usefulness	*Activity 1:* Groupwork to enable stakeholders to discuss and co-produce realistic model scenarios interpret model outputs and confirm their usefulness (heterogeneous grouping) *Activity 2:* Groupwork to explore the importance of model outputs and visualisations which will make WorkHORSE a useful tool for stakeholders (heterogeneous grouping)
4	• dissemination and demonstration of the decision support tool	Stakeholder demonstation of using the model and interpretation of outputs (heterogeneous grouping)

### Evalaution and data collection

Both stakeholders and the modelling team completed questionnaires with open-ended and closed questions to evaluate the co-production process. At the end of each workshop, stakeholders completed stakeholder engagement questionnaires to explore their views and experiences throughout the process. Questions included their reasons for attending the workshops, their expectations and what they gained from attending, and perceived added value of their involvement. The modelling team also completed questionnaires to explore their expectations before the workshops and their experiences thereafter. Questions included the added value of having a series of workshops, the process of co-production, how the decision support tool benefited from stakeholder involvement. We continuously evaluated our patient and public involvement with our lay advisers throughout the project via meetings and email in order to assess impact and to identify areas for improvement in lay adviser involvement. At the end of the project, the modelling team, stakeholders, and lay advisers were emailed a final questionnaire, tailored accordingly, to identify their overall experience of the project and co-production process.

### Thematic analysis

The qualitative information obtained from the questionnaires and notes from meetings with the lay advisers was analysed using the principles of thematic analysis as described by Braun and Clarke (2006) [22]. Familiarisation of the data was carried out, reading through all of the data and generating initial codes based upon the responses to the open questions. These were then grouped into meaningful categories and further searched and reviewed for themes. The responses were then categorised into a sufficiently small set of broad categories, which were then coded and subsequently indexed.

## Results

The series of four workshops were delivered at six monthly intervals across two years (February 2018 to October 2019). Thirty stakeholders participated in the workshops, of which 15 attended two or more workshops. Stakeholders represented the local, regional and national perspective, and included attendees from LAs, CCGs, GPs, Academia, Public Health England and third sector organisations (including NICE and British Heart Foundation).

### Developing a Foundation for Effective co-Production

Stakeholders rated workshop 1 at mean = 4.36, median = 4, range 3–5 (1 being poor, 5 being excellent).

### Expectations of the co-production process

Most stakeholders indicated their anticipation at being able to learn about the WorkHORSE tool, the research process in tool development, and having the opportunity to actively contribute. They also saw their knowledge, expertise and user perspective as potentially contributing to the components of the tool, to ensure that it was user-friendly and relevant to the end-user. Many stakeholders were enthused at the prospect of having a valuable tool which could lead to more effective and equitable health checks delivery. Typical comments included:

***SH2–2*** “*To be included in creating a benefiting tool for the NHS health check programme.”*

***SH12–2*** “I *think it is a potentially hugely valuable tool that could help local areas design programmes which would make them less resistant to universal delivery.”*

Some stakeholders commented how the process could be improved: include more LA and CCG stakeholders as they are key in making the decisions; have an example of the impact_NCD_ model [23] so stakeholders could place what we mean by ‘model’; have more time as it was superficial to truly develop ideas synergistically, challenge them and the associated underlying assumptions in the time allocated; have a sharepoint site to bring tools and documents together; and a wider ‘virtual’ group to gain views electronically.

This was the first time the modelling team had engaged with stakeholders regarding tool development. Prior to Workshop 1, the team expectations of stakeholder’s contribution were mixed. Responses indicated that while they were hoping for some useful and innovative engagement, their was unfamiliarity with the process of stakeholder engagement and what could potentially be achieved.

***M4. “****I think that I was expecting them to provide general ideas on scenario building features, but I wasn’t really expecting them to understand modelling details at the required level. My expectations were more about participation, being able to engage them in a fruitful and useful discussion.”*

Although initially there was apprehension about the process of engaging with stakeholders, the team found Workshop 1 exceeded their expectations and provided added value to the tool development.

***M2. “****Yes, stakeholders were very engaged and came up with lots of ideas. I was particularly pleased with their enthusiasm and interest to participate...****.*** *I think that as the first interaction with them, their understanding was better than I thought, as exemplified by the suggestion of the Best Practices Templates Tool.”*

The modelling team were able to reflect upon their usual process and approach to model building. Usually, decision support tools would be developed with little consultation apart from internal colleagues, and the possibility of discussion with modelling peers.

***M3. “****The modelling team would have made all the decisions without formal external input. After the end of the project, the users, including current stakeholders, would be able to provide feedback; but by then we would have no resources and less flexibility to react to their feedback.”*

### Co-production as a process for tool development

Stakeholders rated workshop 2 at mean = 4.5, median = 5, range 3–5 (1 being poor, 5 being excellent).

By Workshop 2, stakeholders expected the decision support tool to have progressed as a result of their input during Workshop 1. Stakeholders were eager to see a prototype of the tool and how stakeholders were contributing to the tool development.

***SH4–2*** *“To see progress and how engagement with stakeholders had contributed to that progress”.*

***SH9–2*** *“To see a prototype of the tool and how the last workshop has shaped developments so far and inform next steps”.*

Stakeholders expressed greater insight and understanding of what the model would include and how it would work. Furthermore, the opportunity to network with other stakeholders provided the opportunity to gain different perspectives regarding what to include in the model, and how it would be used by various end-users.

***SH3–2*** *“I was keen to see how the model had progressed and how it could be used to produce various scenarios to potentially inform commissioning decisions. It was also a great opportunity to network with people from other areas and organisations”.*

There was a consistent theme of co-production leading to a tool that would be relevant to the end-user and stakeholders being able to provide “real-world experience” related to actual work practices, range of different perspectives and expectations of outputs.

***SH12–2*** *“Massive value – it’s been fascinating to watch academics extract from ‘real-world users’ the information they need to make the tool truly ‘useable’. If the project is to have a tangible outcome (the model tool) it will only be used if the end-users have had an input and ensured it is relevant to them”.*

The perceived value of co-production in model development was a continuous theme that became increasingly highlighted by the stakeholders as the workshops progressed, especially in ensuring relevance for end-users.

***SH1–3*** *“To continue supporting the development of the tool and ensure it caters to the needs of localities that are not pushing boundaries of health checks, and to help ensure we end up with a product that’s going to work on the ground.”*

One participant commented that we needed to know how those most “anti- “NHS Health Checks (i.e. senior leaders not convinced about universal delivery) could use the tool to inform approaches to delivery that would work for their local populations.

Researcher feedback after the delivery of Workshop 2, found the modelling team perceiving co-production as providing validation for the decision support tool being developed and reassured that its development would be of relevance to the end-user. Co-production ensured that all aspects relevant to the end-user were being considered, not just what the development team thought would be required.

By adopting this approach, the end-users would not only have a decision support tool tailored to their needs but would have an in-depth understanding of the process involved in achieving the end product. Likewise, the modelling team had a greater insight into why certain scenarios and outputs were necessary.

***M1.*** *“To make our research meaningful and helpful. To help us on focusing on what is really important for decision makers.”*

***M4.*** *“First and foremost, transparency. Most modelling exercises are opaque.... Our approach put them at the centre of the model, responding to their needs, getting them engaged so that they help disseminate the work once it is finished and be local champions for it.”*

The modelling team saw stakeholders as being able to contribute not only to what was required for the decision support tool to be useful but also what should be excluded, thus making the tool more refined and fit for purpose. Specifically, they welcomed stakeholder contributions in terms of the required inputs, outputs and the graphic user interface.

***M3. “****I expect with their contributions to make the GUI (Graphic User Interface) useful and more intuitive for the users. I also hope to identify which model outputs are more useful to them so I can make them more easily accessible in the GUI.”*

### Consolidation of the decision support tool via co-production

Stakeholders rated workshop 3 at mean = 4.6, median = 5, range 4–5 (1 being poor, 5 being excellent) with all participants stating the workshop achieved its objectives.

Many stakeholders attended Workshop 3 in order to observe how the model had evolved from Workshop 2, and understand how the model would work, especially in terms of outputs.

***SH3–3*** *“To further develop the tool in a positive, energetic, interactive workshop.”*

***SH11–3*** *“To see the next iteration of the tool. See how learning from the previous workshop has been used. Understand more about sustainability and future plans for the tool.”*

Stakeholders also commented upon the added value of their involvement in the series of workshops. It provided them with more confidence in the tool, as they had observed and contributed to its development. Stakeholders’ comments indicated they felt the iterative workshop process for model development was beneficial for both them and the modelling team.

***SH3–3*** “*Genuine proof these workshops and communications- in- between have impact – mixed model and functionality now built in, which is marvellous. A better understanding of reality of delivery for those on the ground.”*

***SH5–3*** *“Awareness that previous comments have been taken into account, and valuable insight and understanding of the tool, its benefits and capabilities.”*

All stakeholders were very positive regarding the advantage of having a series of workshops as opposed to one workshop. Most importantly, they saw it as an opportunity to learn about and reflect upon the tool’s capacity, usage and usefulness as an end product.

***SH2–3*** *“Huge! It would be too much to take on over one day. Division months between workshops provided the opportunity to reflect and think of questions.”*

***SH8–3*** *“You end up with something truly coproduced, doing what people need it too. I worry this is not the case with other things we’ve commissioned development of recently.”*

***SH10–3*** *“Greater clarity and more sophisticated understanding of subsequent iterations of the model. The group was more aware of the detailed issues having attended previous workshops. More informed and detailed discussions.”*

Some stakeholders commented how the process could be improved: to practice more scenarios; to allow more time for discussion; and increase representation from LAs and CCGs.

The third Workshop was a culmination of the co-production process. The modelling team felt it provided an opportunity to refine the decision support tool, achieve consensus and have endorsement of the tool that had been created through the series of workshops.

***M2.*** *“Keep participants on board with the co-production process. Getting feedback before the interface is completed.”*

***M3.*** *“Reassurance that we are travelling to the right direction. At that stage of the project there was still time to improve the fundamentals if necessary.”*

***M4.*** *“Because of the success of the experience, we gained valuable feedback re the user experience with the model, good discussions regarding the complexity and usefulness of the model, and very useful conversations on how the real LA setting in terms of analytical and modelling skills set can be enhanced by the model interface. This will be invaluable for the final design of the user interface.”*

Furthermore, it was felt the stakeholders added dimensions to the tool that would not have been identified by the modelling team alone.

***M3 “****There were many small additional improvements. Most of them very smart and useful that I would have never thought by myself.”*

***M4*** *“. … particularly in how to help the user through the interface to understand some of the concepts and outputs of the model.”*

Having co-produced the model with stakeholders, the modelling team increased confidence in the decision support tool that its was being built. They have reassurance and endorsement from end-users that what has been created will be “fit for purpose”.

***M2*** “*I am really pleased with how the model is looking. It is better than I thought it would be.”*

***M3*** *“I am now confident that the WorkHORSE model may fulfil its purpose to be useful and support policy makers to make better decisions …*”.

***M4 “****I am extremely pleased in viewing in action the principle of co-production. Features suggested in WS2 and implemented and demonstrated in WS3, providing an opportunity to iterate and incrementally improve the usefulness of the model.”*

### Demonstrating proof of concept

Stakeholders rated workshop 4 at mean = 4.4, median = 4, range = 4–5 (1 being poor, 5 being excellent).

Stakeholders attended the final workshop in order to support the development of the model and to contribute to its final iteration.

***SH 4–4 “****To complete the participation in this programme and activity”.*

***SH 8–4*** *“To contribute to stakeholder discussions supporting the development process of this model”.*

Stakeholders appreciated the opportunity to observe and discuss the tool with other stakeholders from different organisations and localities. They commented on the progression of the model and welcomed the opportunity to observe the final version and how it could be used in practice.

***SH 1–4 “****Really great understanding around the tool/data/the art of the possible”.*

***SH 4–4 “****Better understanding of how the model will support me around future decisions for health checks”.*

***SH 8–4 “****Having different perspectives and needs from other stakeholders, seeing the progress and development of this model. Learning the capability of the new tool and how it can be applied”.*

Stakeholders expressed satisfaction with the decision support tool presented to them. They were enthused at the prospect of having a tool that would provide them with various scenarios and being able to demonstrate the capabilities of the NHS Health Checks programme at various levels.

***SH2–4*** *“Opportunity to use the model to show impact of various scenarios which wasn’t available before”.*

***SH4–4*** *“Being able to demonstrate HC effectiveness/HC programme evolving/cost effectiveness of HC is still possible”.*

***SH8–4*** *“The new tool that will be publicly available will provide valuable information of the NHS Health Check at both national and local levels. It is also brilliantly flexible for all types of users in planning, managing and monitoring their local provision”.*

The modelling team valued the opportunity to demonstrate to stakeholders, and for stakeholders to demonstrate to their peers, how the decision support tool had been directly informed by the co-production process. Thus, resulting in a product that would be user-friendly.

***M2 “****Show the tool and how we have responded to stakeholder input into the project”.*

***M3 “****To allow the stakeholders demonstrate the use a working version of the model and to get final comments and suggestions from them”.*

Expectations were met in terms of the *“lively and interesting conversations”(*M1) held between stakeholders and stakeholders and the team. Having workshop 4 enabled the modellers to demonstrate “proof of concept” (M1) and being able to *“debrief stakeholders”* (M2). M3 commented upon how one of the stakeholders tool demonstrations provided new ways of thinking for the modellers: “*… they used the model in a way I haven’t previously thought of. I found this exciting”.*

The process of co-production was deemed a success by the modelling team, with the right mix of stakeholders participating and their views incorporated into tool devleopment. Although one modeller commented, input from practice nurses may have been useful (M2). Also, M1 commented: “*… Of course there is much more to do, as key aspects to be contemplated in the Implementation plan might benefit from more interactions, but sadly we are not funded to do that work.”* This comment was reiterated in relation to changes they would have made to the process of co-production.

Moving forward, all the modelling team felt that model development was only one component of enabling stakeholders to use the tool to inform decisions. Stakeholders would require training and support to ensure successful implementation in the workplace.

***M1 “****Develop their own use cases and modify edit the tool for that purpose We build the tool with that flexibility, so it will be the ultimate proof of concept.”*

***M2 “****Some funding for training and ongoing support”.*

***M3 “****We produced a prototype. Now we need the production pipeline and the training”.*

### Lay advisers feedback

The lay advisers felt valued in their involvement in the project. They perceived their role as acting as public consultants and translators of information for a wider audience and as advocates adding value from the public perspective and observing the “return on investment” for the public funding provided to the project via the research body.

Their involvement in the writing of the research proposal was highlighted as a positive approach to co-producing with the public:

***LA 1*** “*… beginning was excellent in terms of involvement. I loved commenting on the bid. The first meeting was taking our views and making a key contribution to how the bid would look … although it felt a bit over my head, we thought it was looking at Health Checks, not specifically modelling. It took time to figure out the idea of modelling … .but I did enjoy it.”*

However, as illustrated by the comment above, the lay advisers felt their involvement was inhibited by the nature of the project. They commented that ***LA 2*** “*the project was quantitative and we look more into the qualitative … PPI is important because it influences the care standard of what the patient is receiving.”*

Conversley, they did identify their valuable role in dissemination, in order to ensure that the research findings reached the public realm, including co-producing summaries of the research for publication in, for example, local government newsletters.

## Discussion

### Summary of main findings

We believe this to be an innovative project in the field of developing a decision support tool using the principles of co-production and evaluating the experiences and reflections of those involved in the process. Studies have reported upon co-production as a method of increasing user involvement, primarily in the form of exploratory studies [24, 25] or case study [26–31] reviews. However, there is a dearth of literature relating to the combined experience of a team of modelling researchers and stakeholders on iteratively co-producing a simulation decision support tool.

Stakeholders experiences were positive overall. They felt valued and commended the involvement of practitioners. Major reasons for particpation included being able to influence the development of the tool and gaining insight and understanding of what the tool could include and how it would work in practice. They appreciated the iterative process involving a series of workshops which provided opportunities for them to learn about and reflect upon the model’s capacity, usage and usefulness. We have previously reported on the engagement with the stakeholders in workshop 1, where we aimed to explore the NHSHCP in terms of what is working well, less well and future hopes, and explore features to potentially include in a useful decision-support tool for stakeholders. The stakeholders demonstrated their commitment to NHSHCP whilst highlighting the perceived requirements for enhancing the service and discussing how the decision-making tool would be instrumental in this process. Their suggestions for improvement informed subsequent WorkHORSE workshops and model development [32].

The modelling team saw the process as an opportunity for developing a common language and trust in the end product and ensuring the support tool was transparent. The workshops acted as a reality check, ensuring model scenarios and outputs are relevant and fit for purpose.

This was the first time that our research team had involved lay advisers in a research project involving the development of a modelling tool. As a team we learnt the added value of having a lay perspective on the project being delivered and consideration of how we can improve our lay involvement in the process of co-production. Future projects could be enhanced by having PPI input at the research proposal development stage, together with a strategy outlining definitive roles for the project duration. There is a need to be aware that lay advisers are members of the public and the academic environment can be initially daunting. Time is required to establish relationships and to enable lay advisers to familiarise themselves with the academic environment as well as the project itself.

### Linking to prior studies

Jun et al. (2011) reported a similar methodology for the development of a modelling simulation tool for health care management. Albeit they did not evaluate the engagement process, they believed such an approach would enable health care modelling consultants and researchers to collaboratively inform future models to meet the diverse needs of various health care clients [33]. Key features of the WorkHORSE co-production process were: a) having on-going dialogue between the modelling team and stakeholder participants and b) ensuring stakeholder feedback was an integral part of the decision support tool development, thereby ensuring the final tool would be valid and relevant for the NHS HCP. This is supported by Tabriz et al. (2019) [31], who describe the development of a logic model framework for a healthcare organisation–university-based research partnership for delivery system sciences. Key elements to success included pragmatic and responsive researchers and knowledgeable stakeholders; having shared learning and problem-solving opportunities; and transparent and joint decision-making.

The basic requirements to co-produce in research is the willingness and capacity of the participants to be involved, and for the researchers to appreciate the participants’ contributions [34]. Writers have previously commented that co-production in research “interferes” with conventional practices of researchers [13]. This is a major issue in the development of dynamic simulation tools, where researchers rarely consult with the end-user. Including participants with no research background in the decision-making requires major changes in the researcher’s role and the power-relationship between them and the participants (Pohl et al., 2010) [17]. In this study, the stakeholders demonstrated a commitment to developing a decision support tool which would benefit the delivery of the NHS HCP. The modelling team had no previous experience of engaging with stakeholders in developing a decision support tool and there was initially some uncertainty about the added value of involving stakeholders. However, it soon became apparent the benefit of stakeholder involvement and the team embraced the process of co-production.

Oliver [18] highlights the various costs of co-producing research with stakeholders. These include the practical costs (e.g. arranging venue, travel, researcher time and resources), personal costs to researchers (e.g. increased interpersonal conflict), costs to research (e.g. time and effort to manage relationships), costs to stakeholders (e.g. time away from their day job). Our experience endorses many of these. The series of workshops entailed a substantial amount of preparation, planning and choreographed delivery, with regular team meetings. Also, we needed to ensure the location was accessible to stakeholders and have the technology for demonstrating and developing the tool. Recruitment of the stakeholders was only the beginning of the process, maintaining and building upon initial interest and commitment took time and required an understanding of their profession, what they brought to the workshops, and what they wanted from the decision support tool. As a research team, we were also conscious of the added pressures upon the modellers to build the decision support tool based upon the iterative process. It was a change to the traditional approach of no or limited consultation, resulting in additional time required. From our experience, we encourage the use of co-production. However, co-production should not be taken on lightly, serious consideration should be given to the added benefit for the outcome of the research by co-producing. There needs to be a supportive and constructive team, with defined yet collaborative roles. Substantial time and commitment need to be allocated to the process. There also needs to be clear aims and objectives and outcomes for the workshops themselves and the overall project.

### Strengths and limitations

The strengths of this study are the use of a systematic and transparent process [20] to coproduce the priorities for the decision support tool. The series of four iterative workshops provided an ideal opportunity to engage with a broad range of stakeholders. There was opportunity for individual opinions to be expressed via writing down views before group discussion while engaging in group work activities encouraged group consensus. The involvement of lay members of the public also contributed to decisions about project development, delivery and dissemination.

There are limitations. Firstly, although stakeholders from a variety of organisations and backgrounds attended, most of the stakeholders were unable to attend all four of the workshops leading to potential bias. For example, some stakeholders only attended Workshop 1 and 3. However, this was addressed by providing a “catch up” session immediately prior to the main workshop for previous non attendees. We had a good representation of stakeholders from all levels (national, regional and local) and are confident we captured a cross-section of views. Secondly, although stakeholders represented a cross-section of local and national organisations, participants were not always represented by the end-user of the decision support tool. Future studies should include more end-users who may use decision tools (e.g. public health analysts). Furthermore, stakeholder feedback was predominantly supportive, suggesting participants may have been bias to those who were eager for a modelling tool to be successfully developed and implemented to support the commissioning of the NHS HCP. Future projects should ensure a more diverse group of stakeholders (e.g. sceptical of the use of modelling tools for NHS HCP delivery). Thirdly, lay members overall felt valued in being involved in the project however the nature of the project (development of a computer decision support tool) at times meant that lay members did not feel able to fully contribute to the co-production process. This might require further thinking on how to effectively involved lay perspectives in modelling when the public is not the intended end-user. Future projects need to ensure that lay members are better informed about the nature of the research and feel confident in their ability to contribute.

## Conclusions

The WorkHORSE dynamic simulation tool was developed to provide decision-makers and practitioners with a web-based decision-support tool to help identify the most effective, cost-effective and equitable interventions for the NHSHCP. Computational modellers rarely consult with end-users when developing tools to inform decision-making. The added value of involving stakeholders in the co-production of tool development enabled productive and valuable dialogue, provided valuable learning about potential problems in practice, and supported consensus building for effective end-use. The resulting level of engagement resulted in modellers producing a “real-world” operational tool, with the capacity to test a large range of scenarios to determine their likely short-term and longer-term impacts. Likewise, stakeholders have increased confidence in the decision support tool’s development and applicability in practice, with a robust basis for decisions on the delivery of the NHSHCP.

## Supplementary information

**Additional file 1.**

## Data Availability

The datasets used and/or analysed during the current study are available from the corresponding author on reasonable request.

## Abbreviations

BMA: British Medical Association
CCGs: Clinical Commissioning Groups
CVD: Cardiovascular Disease
GMB: Group Model Building
GPs: General Practitioners
LAs: Local Authorities
M: Modeller
NHSHCP: National Health Service Health Checks Programme
NICE: The National Institute for Health and Care Excellence
SH: Stakeholder
WorkHORSE: working Health Outcomes Research Simulation Environment
WS: Workshop
