# Supplementary material for: Evaluating stakeholder involvement in building a decision support tool for NHS health checks: co-producing the WorkHORSE study
Source: BMC Med Inform Decis Mak. 2020 Aug 10;20:182. doi: 10.1186/s12911-020-01205-y (PMC7418313; doi:10.1186/s12911-020-01205-y)
Supplement: Supplementary file 1 — Additional file 1. [file 12911_2020_1205_MOESM1_ESM.docx]

**APPENDIX 1 - STAKEHOLDER ENGAGEMENT WORKSHOP 1**

***Objectives:***

1. *To enable stakeholders to meet and develop a shared understanding of the current implementations of the NHS Health Check Programme (NHS HCP)*
2. *To identify what is working well and not so well, and future hopes for the NHS HCP*
3. *To explore what features and specifications will make WORKHORSE a useful tool for stakeholders.*
4. *To identify stakeholder’s expectations of the WORKHORSE health checks project*

***Outcomes***

1. *To have built a mutual working relationship between stakeholders and the research team.*
2. *To have a better understanding of the health checks process for scenario building and model development.*
3. *To have generated rich and valuable feedback to inform model development and the second workshop.*

**workHORSE Health Checks Model Building - Workshop 1 Plan**

10.00 – 10.20 **Arrival and Coffee (20 mins) (Registration – including name/organisation badges and collect/complete consent forms)**

10.25 – 10.35 **Welcome (10 mins)** (inc. overview of the day, why they have been invited, what they will get out of attending) **and** **Introductions** (Team Members/Lay Advisers – who we are and what are role will be during the workshop), housekeeping (fire alarms/toilets) ground rules and overview of the workshop

10.35 – 10.45 **Ice Breaker (10 Mins)** All attendees (inc. team members/lay advisers) divide into twos to introduce themselves, where from, occupation/background, and “Find out one thing you have in common outside of work”

10.45 – 11.15 **(30 Mins)** Each **participant provides feedback** on behalf of the participant they have been talking to

11.15 – 11.30 **Brief overview of WORKHORSE (Slides)**

1. Description of project  **(5 mins)**
2. Stakeholder engagement **(5 Mins)**
   1. Workshop purposes and structure
3. Questions relating to presentations **(5 mins)**

11.30– 11.40 **COFFEE BREAK**

11.40– 11.50 **Presentation of current WORKHORSE (Slides) (10 mins):** Liverpool Health Checks Model

11.50 – 12.00 **Questions/Discussion (10 mins)** relating to Presentation of the current WORKHORSE Liverpool Health Checks Model **(Objectives 1)**

12.00 – 12.25 **(25 Mins)** **Individually (5 minutes) then in Groups of (3/4) (20 minutes) (Slides explaining activity)** NHS Health checks decision making: what is working well/not so well and future hopes **(Objective 2) *(see Activity 1 for instructions)***

12.25 – 12.50 **(25 mins)** **Groups feedback then whole group discussion**

12.50 – 13.00 **(10 mins)** **Summary of themes and outcomes** emerging from activity session

***Ask to complete and hand in expenses form during lunch break***

13.00 – 13.40 **LUNCH and networking**

13.40 – 13.50 **Welcome back Activity to get participants motivated (2 mins),** summary of morning (key points), introduction to afternoon **(8 mins)**

13.50– 14.15 **(25 mins) Individually (5 mins) then in Groups (3/4) (20 mins) (Slides explaining activity)** How to make WORKHORSE a useful tool **(Objective 3) *(see Activity 2 for instructions)***

14.15 –14.40 **(25 mins)** **Groups** **feedback then whole group discussion (25 mins) (Objective 3)**

14.40 – 14.45 **(5 Mins)** **Summary of themes and outcomes** emerging from activity session

14.45 – 15.15 a. **Stakeholders summing up and expectations**  Ask everyone, based upon their experience of workshop 1, to write down their expectations for the health checks project and added value of having stakeholder involvement **(10 mins)**. Go around each table and ask for 1 or 2 participants to provide their feedback **(10 mins)** **(Objective 4)**.

b. Completion of **evaluation form (10 mins)**

c. Reminder to complete and hand in expenses form to Fran before leaving.

15.15 – 15.30 **(15mins)** **Summary/Overview of day (10 mins) (Slides);** what happens next (i.e. model development/ overview of workshop 2/continued feedback via website);

- *Sum up and review agenda.* Restate the major points that the workshop covered and revisit the agenda to identify any areas left uncovered; summarise workshop outcomes; inform how the results will be used; what is expected from them moving forward
- *Next steps:* Inform that the workshop feedback and actions will be collated and circulated to all participants (encourage them to share with colleagues) and provide comments if they wish. Also, encourage participants to email after the workshop if they have any further thoughts and ideas they wish to share.

**(5 mins)**

**Any questions**

**Thank you for attending**

**ACTIVITY 1 (Objective 2)***To identify stakeholder’s expectations of the WORKHORSE health checks project; To identify what is working well and not so well and future hopes for the NHS Health Checks Programme (NHS HCP)*

**Purpose:** To elicit what is working well and not so well and future hopes for the NHS HCP

**Time:** 25 minutes for the activity; 25 minutes feedback from individuals and group discussion

**Materials:**

1. Flipchart paper with headings (*what is working well, not so well and future hopes)* for each table of participants
2. Three different colours of post it notes for each participant
3. Back felt tip pens for each stakeholder
4. Masking tape
5. Tape recorder to record individual/group feedback/discussion

**Outputs:**

List of stakeholder’s comments regarding what is working well and not so well and future hopes for NHS HCP.

**Roles:**

1. Facilitator with knowledge of the topic
2. Scribe/recorder to document the session

**Steps:**

1. Participants are given several sheets of paper in each colour. The facilitator explains that they will be writing what they believe works well and not so well and future hopes relating to NHS HCP and then sharing them with the group.
2. The facilitator states which colour post it note represents working well; not working well; future hopes, and to stick responses onto the appropriate piece of flipchart paper (with headings)
3. Stakeholders have 25 minutes for the whole task (5 minutes to write down individual thoughts, 20 minutes to discuss with other group members)
4. Each stakeholder reads out one aspect working well, one not so well and future hope for NHS HCP. After each participant has shared once, facilitator opens the floor to all participants for any further comments about what is working well and not so well, challenges and future hopes and opportunity for discussion. (25 minutes)
5. Facilitator then tries to identify some of the themes and summarises. (10 minutes)
6. Scribe/recorder records all comments regarding what is working well and not so well and future hopes in the session notes.
7. *ALL flipchart paper to be collected from each table and either stuck up at the back of the room or laid on tables at the back of the room – stakeholders are encouraged to add additional comments at any time to the flipchart papers.*

**ACTIVITY 2 (Objective 3)** *To identify how to make WORKHORSE a useful tool*

**Purpose:** To elicit what features/specifications will make WORKHORSE a useful tool for the stakeholders.

**Time:** 25 minutes for the activity; 25 minutes feedback from individuals and group discussion

**Materials:**

1. Flipchart paper with heading (*features/specifications that will make WORKHORSE a useful tool)* for each table of participants
2. One colour of post it notes for each participant
3. Back felt tip pens for each participant
4. Masking tape
5. Tape recorder to record individual/group feedback/discussion

**Outputs:**

List of stakeholder’s comments regarding what will make WORKHORSE a useful tool

**Roles:**

1. Facilitator with knowledge of the topic
2. Scribe/recorder to document the session

**Steps:**

1. Each participant is given post it notes, and each table will have flipchart paper. The facilitator explains that they will be writing what they believe will make WORKHORSE a useful tool for them, answering the question: From your current position and level of involvement in NHS HCP, What specifications should the WORKHORSE tool have in order to facilitate your work/decision process?
2. The facilitator will ask participants to write only one *feature/specification* per post it note. With all post it notes to be stuck onto the flipchart paper on their table.
3. Stakeholders have 25 minutes for the whole task (5 minutes to write down individual thoughts, 20 minutes to discuss with other group members)
4. Each table reads out maximum three specifications for WORKHORSE tool. After each table has shared once, facilitator opens the floor to all participants for any further comments about what will make WORKHORSE a useful tool. (25 minutes)
5. Facilitator then tries to identify some of the themes and summarises. (5 minutes)
6. Scribe/recorder records comments regarding what will make WORKHORSE a useful tool.
7. *ALL flipchart paper to be collected from each table and either stuck up at the back of the room or laid on tables at the back of the room – stakeholders are encouraged to add additional comments at any time to the flipchart papers if they wish.*
